# *MEF2C* rs190982 polymorphism with late-onset Alzheimer's disease in Han Chinese: A replication study and meta-analyses

**DOI:** 10.18632/oncotarget.9819

**Published:** 2016-06-03

**Authors:** Shan-Shan Tang, Hui-Fu Wang, Wei Zhang, Ling-Li Kong, Zhan-Jie Zheng, Meng-Shan Tan, Chen-Chen Tan, Zi-Xuan Wang, Lin Tan, Teng Jiang, Jin-Tai Yu, Lan Tan

**Affiliations:** ^1^ Department of Neurology, Qingdao Municipal Hospital, School of Medicine, Qingdao University, Qingdao, PR China; ^2^ Department of Emergency, Qingdao Municipal Hospital, School of Medicine, Qingdao University, Qingdao, PR China; ^3^ Department of Geriatric, Qingdao Mental Health Center, Qingdao, PR China; ^4^ College of Medicine and Pharmaceutics, Ocean University of China, Qingdao, PR China; ^5^ Department of Neurology, Nanjing First Hospital, Nanjing Medical University, Nanjing, PR China

**Keywords:** Alzheimer's disease, MEF2C, rs190982, association study, meta-analysis, Gerotarget

## Abstract

The myocyte enhancer factor (*MEF2*) family of transcription factors plays a vital role in memory and learning due to its functions in regulating synapse number and reducing dendritic spines. Myocyte enhancer factor 2 C (*MEF2C*) is regarded as modulator of amyloid-protein precursor (APP) proteolytic processing, in which amyloid-β (Aβ) is produced. A common single nucleotide polymorphism (SNP, rs190982) in *MEF2C* gene was identified to be related to late-onset Alzheimer's disease (LOAD) in Caucasians in a large meta-analysis of genome-wide association studies (GWAS). Here, we recruited unrelated 984 LOAD patients and 1348 healthy controls matched for gender and age to ascertain whether the rs190982 polymorphism is related to LOAD in Han Chinese. No difference in the genotype and allele distributions of the *MEF2C* rs190982 polymorphism was found between LOAD cases and healthy controls (genotype: *P* = 0.861; allele: *P* = 0.862), even after stratification for *APOE* ε4 allele as well as statistical adjustment for age, gender and *APOE ε4* status. Furthermore, the meta-analysis in 4089 Chinese individuals did not detect the association of rs190982 within *MEF2C* with the risk for LOAD (OR = 1.03, 95%CI = 0.90-1.18). Overall, the current evidence did not support the relation between rs190982 polymorphism within *MEF2C* and the LOAD risk in Northern Han Chinese.

## INTRODUCTION

Alzheimer's disease (AD) is defined clinically by gradual decline in cognition and function as well as loss of memory and pathologically by extensive neuronal and synaptic loss, intracellular neurofibrillary tangles and extracellular amyloid-β (Aβ) peptides deposition [1, 2]. Genetic variations are widely considered to have vital roles in the progress of AD [3]. Mutations in β-amyloid precursor protein (*APP*), presenilin 1 (*PSEN 1*) and presenilin 2 (*PSEN 2*) genes contribute to the uncommon early-onset form of the disease (onset < 65 years; EOAD) [4]. As to the more common late-onset AD (onset > 65 years; LOAD), only the *APOE ε4* has been consistently identified to be a risk factor of the disease [5]. In addition, some GWAS have identified 21 additional genetic susceptibility factors for LOAD including bridging integrator 1 (*BIN1*), inositol polyphosphate-5-phosphatase D (*INPP5D)*, triggering receptor expressed on myeloid cell 2 (*TREM2*), *NME8* (encoding NME/NM23 family member 8), the clusterin gene (*CLU*), protein tyrosine kinase 2β (*PTK2B)*, desmoglein 2 (*DSG2)* and others [6]. However, the known LOAD genes mentioned above are not sufficient to account for the total genetic variance, indicating additional risk genes or locus remain to be discovered [7].

Among the known genetic factors, *MEF2C* gene is supposed to be connected with synaptic function that is altered in AD. Transcription factors *MEF2* family (*MEF2A-D*) modulate the structural and synaptic plasticity underlying memory formation [8] and can regulate the synapse number in the hippocampus in which its activation restrains the development of dendritic spines, highlighting its important roles in memory and learning [9]. A recent work revealed that the overexpression of *MEF2* function impeded the learning-induced increases in spine density and impaired memory formation *via* an Arc-mediated reduction in the surface expression of the GluA2-AMPA-type glutamate receptor [10]. In brain, *MEF2C* is highly expressed in the regions which are closely related with learning and memory such as dentate gyrus, frontal cortex, entorhinal cortex, and amygdala [11]. On the other hand, *MEF2C* may be involved in the inflammatory process altered in AD *via* the regulation of microglia proliferation [11]. In addition, *MEF2* seems to play an important role in APP-mediated anti-apoptotic neuroprotection [12], and *MEF2C* is identified as a regulator of APP proteolytic process in which Amyloid-β (Aβ), one central factor to initiate AD pathogenesis, is produced [13]. Recently, a large meta-analysis has confirmed that the SNP rs190982 polymorphism within MEF2C on chromosome 5 acted as a protective factor for AD dementia (OR = 0.93, 95%CI = 0.90-0.95, *P* = 3.2×10^−8^) in the Caucasian population [14]. Similarly, the association between the *MEF2C* gene rs190982 polymorphism and AD was also confirmed in a large Spanish sample (OR = 0.885, 95%CI = 0.811-0.966) [15]. Furthermore, another two studies replicated the association in China Han. Regretfully, they failed to reveal any association of rs190982 polymorphism in *MEF2C* gene with AD susceptibility in our Han Chinese cohort [16, 17]. Here, we conducted a case-control study containing 2332 individuals to clarify whether the selected SNP on *MEF2C* gene has an association with LOAD in Northern Han Chinese population.

## RESULTS

We enrolled 2332 ethnic Northern Han Chinese subjects including 984 subjects (42.20%) with probable LOAD and 1348 healthy control subjects (57.80%). Table 1 lists the demographic and clinical characteristics of the recruited subjects. LOAD cases and control group were matched in terms of age (*P* = 0.242) and gender (*P*= 0.088). As expected, AD patients showed significantly lower MMSE scores (11.99±6.198) than controls (28.99±0.124; *P* < 0.001). The *APOEε4* allele frequency was also significantly different between patients and control subjects, and was a risk factor of LOAD (OR = 2.409, 95%CI = 1.960-2.962, *P* < 0.001) (Table 1).

**Table 1:** The characteristics of the study population

	AD (*n* = 984)	Controls (*n* = 1348)	*P*	OR(95%CI)	
Age, years; mean±SD	75.15±6.079^a^	75.46±6.463^b^	0.242		
Gender, *n* (%)			0.088		
Male	406 (41.3)	604(44.8)			
Female	578 (58.7)	744 (55.2)			
MMSE score, mean±SD	11.99±6.198	28.99±0.124	<0.001^*^		
APOE ε4 status, n (%)			<0.001^*^	2.409(1.960-2.962)	
APOE ε4 (+)	280 (28.5)	191 (14.2)			
APOE ε4 (−)	704 (71.5)	1157 (85.8)			

Abbreviations: AD, Alzheimer's disease; n, number; OR, odds ratio; CI, confidence interval; SD, standard deviation; MMSE, Mini-Mental State Examination; APOE ε4 (+): subjects who contain 1 or 2 ε4 alleles; APOE ε4 (−): subjects who do not contain ε4 allele.

* *P* < 0.05, significant values.

a Mean age at onset.

b Mean age at examination.

Genotype distributions of the rs190982 was in Hardy-Weinberg equilibrium (HWE) in controls (*P* > 0.05) but was not in the HWE (*P* = 0.02) in LOAD. The genotype and allele frequencies of rs190982 in AD patients and controls in the total sample and after stratification for *APOE ε4* allele were summarized in Table 2. There is no statistical significance between the frequency of the minor allele G in LOAD and that in controls (16.06% *versus* 16.25%). No significant difference was found in allele frequency between LOAD patients and controls (OR = 0.986, 95% CI = 0.842-1.155, *P* = 0.862). Identically, the genotype distribution did not differ significantly between this two groups (*P* = 0.861). Furthermore, multivariate analysis also failed to demonstrate any significant differences between LOAD and controls after adjustment for age, gender, and the *APOE ε4* allele carrier status as shown in Table 3 (Dominant, OR = 1.001, 95% CI = 0.835-1.200, *P* = 0.993; Recessive, OR = 0.737, 95% CI = 0.388-1.397, *P* = 0.349; and Additive, OR = 0.980, 95% CI = 0.830-1.156, *P* = 0.810). Our sample size had over 90% power to detect the moderate difference in allele and genotype distributions between LOAD cases and controls at a 0.05 significance level.

**Table 2:** Allele and genotype frequencies of the SNP rs190982 in total subjects and stratified by ApoEε4 status respectively

rs190982	Total samples			ApoE ε4 carriers			ApoE ε4 non-carriers		
	AD (n%)	Control (n%)	*P*	AD (n%)	Control (*n*%)	*P*	AD (n%)	Control (n%)	*P*
A/A	684(69.51)	936(69.44)	0.861	194 (69.28)	135 (70.68)	0.858	490 (69.60)	801 (69.23)	0.445
A/G	284(28.86)	386(28.64)		78 (27.86)	52 (27.23)		206 (29.26)	334 (28.87)	
G/G	16(1.63)	26(1.93)		8 (2.86)	4 (2.09)		8 (1.14)	22 (1.90)	
A	1652(83.94)	2258(83.75)	0.862	466 (83.21)	322 (84.29)	0.660	1186 (84.23)	1936 (83.67)	0.647
G	316(16.06)	438 (16.25)		94 (16.79)	60 (15.71)		222 (15.77)	378 (16.33)	

Abbreviations: AD, Alzheimer's disease; ApoE ε4 carriers, subjects who contain 1 or 2 ε4 alleles; ApoE ε4 non-carriers, subjects who do not contain ε4 allele.

**Table 3:** Logistic regression analysis of rs190982 polymorphisms

Model	Total sample^a^			ApoE ε4 carriers^b^		ApoE ε4 non-carriers^b^	
	OR(95%CI)	*P*	P for APOE interaction	OR(95%CI)	*P*	OR(95%CI)	*P*
rs190982							
Dom	1.001(0.835-1.200)	0.993	0.734	1.062(0.710-1.588)	0.771	0.986(0.804-1.209)	0.890
Rec	0.737(0.388-1.397)	0.349	0.208	1.441(0.425-4.881)	0.557	0.551(0.243-1.248)	0.153
Add	0.980(0.830-1.156)	0.810	0.534	1.082(0.758-1.544)	0.663	0.954(0.790-1.151)	0.622

Abbreviations: ApoE ε4 carrier, subjects who contain 1 or 2 ε4 alleles; ApoE ε4 non-carriers, subjects who do not contain ε4 allele; OR, odds ratios; CI, confidence interval; Dom, dominant model; Rec, recessive model; Add, additive model.

a Adjusted for age, gender and APOE ε4 status,

b Adjusted for age and gender.

We then explored the effects of the interaction between rs190982 polymorphisms and *APOE* genotype on the risk of LOAD, and no interaction between rs190982 and *APOE* genotype was observed (Table 3). To further investigate whether the presence of the *APOE ε4* allele significantly influenced the effect of *MEF2C* on LOAD, we stratified the data according to *APOE ε4* status. Finally, there were still no differences in the genotypic or allelic distributions between the two groups (Table 2), and rs190982 did not influence the risk for LOAD adjusting for age and gender in *APOE ε4* allele carriers or non-carriers (Table 3).

**Figure 1:**
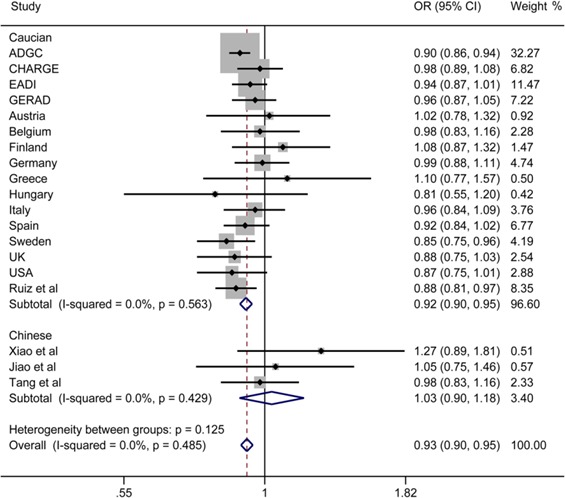
Forest plots for rs190982 in LOAD and healthy controls in 82507 individuals

**Figure 2:**
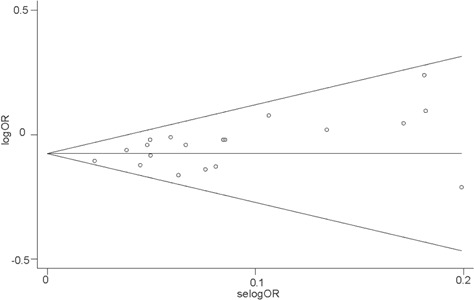
Begg’ funnel plot with pseudo 95% confidence limits

Finally, we performed a meta-analysis about the relationship between rs190982 and LOAD and detected that rs190982 had a protective role in LOAD (OR = 0.93, 95%CI = 0.90-0.95) with no heterogeneity (I^2^ = 0.0%) in the 82507 individuals without ethnic stratification. Then, rs190982 reduced the risk for LOAD in meta-analysis of 78418 Caucasian individuals (OR = 0.92, 95%CI = 0.90-0.95); however, when we combined our data with results from other two studies in China, *MEF2C* (rs190982) was not indicated to be related to LOAD risk (OR = 1.03, 95%CI = 0.90-1.18) (Figure 1). In addition, there is no publish bias in our meta-analysis and the funnel plot is listed (Figure 2).

## DISCUSSION

Myocyte enhancer factor 2 (*MEF2*) family is a member of the transcription factors, which is vital for linking external stimuli to protein production. *MEF2C* is more widely expressed and modulates the development and differentiation of many tissues as well as other various transcriptional events [18]. Gene transcription and protein synthesis are also critical for the long-term memory formation. Individual members of the *MEF2* family can differentially regulate the expression of brain-derived neurotrophic factor (*BDNF*), which performs essential roles in modulating synaptic growth and function [19]. *MEF2* is also a regulator of the activity-regulated cytoskeleton-associated (*ARC*) which is in the controller of memories. As such, *MEF2* remains a key player in memory processes [20]. In addition, *MEF2* decreases spine growth that is critical for the cognition and memory formation and several forms of intellectual disability have been strongly related with the changed spine densities in both animal models and human patients [8, 21]. Moreover, *MEF2* also participates in time-dependent reorganization and context memory consolidation [8]. Interestingly, *MEF2* and an amount of *MEF2* target genes have been implied in several neuropsychiatric and cognitive disorders, which indicate that at least some of the cognitive deficits may be ascribe to the dysfunction of *MEF2*.

Our study did not detect any association of SNP rs190982 polymorphism within *MEF2C* with the development of LOAD in Han Chinese population, even after statistical adjustment for age, gender and *APOE ε4* status in the total sample and stratification for *APOE ε4* status. Similarly, a research with the same aim conducted in China Han also did not declare any significant association between rs190982 and LOAD (*P* = 0.196, OR = 1.27, 95%CI = 0.89-1.81) [16]. Subsequently, Jiao et al. [17] draw the same conclusion that *MEF2C* gene rs190982 polymorphism had no relation with LOAD as well (*P* = 0.798, OR = 1.047, 95%CI = 0.747-1.461). In contrast to our results, Lambert et al. [14] identified rs190982 as a protective factor to the development of LOAD in Caucasian patients in a large-scale GWAS (OR = 0.93, 95%CI = 0.90-0.95). In addition, Ruiz et al. [15] also detected that rs190982 could decrease the risk of AD through multiple test in a large Spanish sample (OR = 0.885, 95%CI = 0.81 1-0.966).

Several factors may be responsible for these discrepancies in these researches. The key one of these factors is genetic heterogeneity in various ethnic populations, such as the difference of minor allele, different minor allele frequency (MAF) and the complexity of the underlying genetic architecture [22]. The MAF of rs190982 on MEF2C is lower in Han Chinese populations than that in Caucasians (0.16% *vs* 0.42%) from the SNP web (http://www.ncbi.nlm.nih.gov/projects/SNP/snp_ref.cgi?rs = 190982). Moreover, genotype distribution of the rs190982 was not in HWE in LOAD group, which may influence the results. In addition, MEF2C rs190982 polymorphism may have adverse effects on the development of AD that likely attribute to difference in sample sizes (Caucasian 74 046 *vs*. Chinese 4089) and unclear specific gene-gene or gene-environment interactions [23]. Furthermore, statistical deviation may have resulted from some other causes including various clinic characteristics of the samples, demographic variables, coexistence of some other unrealized neuropsychiatric change, experiment methods and statistical analyses [24]. Therefore, meta-analysis was performed in the total sample of 82507 individuals, Han Chinese and Caucasian to avoid these possible complex reasons and further explore the associations. The results revealed that rs190982 within *MEF2C* had a positive association with LOAD mainly in Caucasian residents. Although our study failed to replicate any association of the examined SNP with LOAD in the Chinese subgroup, we could not claim that other SNPs of *MEF2C* was not associated with LOAD. Additionally, the renewed genetic sequencing about *MEF2C* may be helpful to find new loci that related to LOAD in the near future.

In conclusion, our outcomes of meta-analysis did not detect any association of *MEF2C* gene rs190982 polymorphism with the risk of LOAD in the North Han Chinese. Further genetic analyses of this locus and the functional regions surrounding *MEF2C* are required to better elucidate the biochemical mechanisms and the interactions with AD. Additionally, larger independent replications both in Han Chinese and other ethnic groups are needed to elucidate the potential role of the locus at *MEF2C* in LOAD pathogenesis because of the genetic variations.

## MATERIALS AND METHODS

### Subjects

In our study, there are 2332 unrelated northern Han Chinese residents in origin from Shandong Province, comprising 984 sporadic LOAD patients and 1348 healthy controls. We screened the qualified AD patients in the Department of Neurology at Qingdao Municipal Hospital and some other 3A-level hospitals. All patients were undergone neuropsychological examination and structural neuroimaging (magnetic resonance imaging and/or brain computed tomography). At least two neurologists together defined a clinical diagnosis of AD based on the criteria of the National Institute of Neurological and Communicative Disorders and Stroke-Alzheimer's Disease and Related Disorders Association (NINCDS/ADRDA) [25]. Never did their first-degree relatives have dementia in their family history. We obtained the information of patients such as age at onset and family history from caregivers. Healthy controls that were matched with patients for age and gender were recruited from the Health Examination Center of each collaborating hospital. Physicians and neurologists confirmed that control subjects were healthy and neurologically normal by following medical history, general examination, laboratory examination, and Mini Mental State Examination (score > 28). Subjects without significant illness such as type 2 diabetes mellitus (T2DM), heart disease, autoimmune disease, stroke, infectious disease, cancer, glaucoma, and atherosclerosis were considered as healthy and neurologically normal in our study [26]. Demographic details of the sample set are exhibited in Table 1. This study was performed after the informed consent of all subjects or legal guardians and with support from the Ethical Committee of Qingdao Municipal Hospital [27].

### Genotyping

We used the Wizard genomic DNA purification kit (#A1125; Promega, USA) to extract genomic DNA from peripheral blood leukocytes of LOAD patients and healthy individuals in accordance with manufacturer's protocol. *MEF2C* polymorphism (rs190982) was genotyped with the method of SNPscan™ kit. This is a patented SNP genotyping technology of Genesky Biotechnologies Inc., based on double ligation and multiplex fluorescent polymerase chain reaction (PCR). Genotyping of *APOE* (rs429358 and rs7412) polymorphisms was performed by the developed multiplex ligase detection reaction (iMLDR) *via* TaqMan genotyping assays on an ABI Prism 377 Sequence Detection System (Applied Biosystems), approved by the Shanghai Genesky Biotechnology Company. GeneMapper Software v4.1 (Applied Biosystems, USA) was used to analyze raw data and the ligation-PCR method was used to determine genotypes for each locus.

### Statistical analysis

Statistical analysis was carried out with the use of SPSS16 software. Hardy-Weinberg equilibrium (HWE) test was performed before the association analysis. Differences in the characteristics of the study population between AD patients and controls were evaluated using Student's *t*-test or the chi-square test. Genotypes and alleles were compared using the chi-square test or the Fisher's exact test. Differences between cases and controls after stratification for *APOE ε4* status were also examined by the chi-square test. We also performed logistic regression to further analyze the relation between the polymorphism and the LOAD risk adjusting for age, gender, and *APOE ε4* status under various genetic models that were defined as 1 (GG+ AG) *versus* 0 (AA) for dominant, 1 (GG) *versus* 0 (AG + AA) for recessive, and 0 (AA) *versus* 1 (AG) *versus* 2 (GG) for additive. In addition, we also evaluated the effect of the interaction between rs190982 and *APOE ε4* on the risk for LOAD in logistic regression models. The P value, odds ratios (ORs) and 95% confidence intervals (CIs) were computed to estimate the association between SNPs and AD. *P* < 0.05 was defined as the statistical significance level. Estimation of the statistical power was assessed with STPLAN 4.3 software.

In order to identify all articles that explored the association of the *MEF2C* rs190982 polymorphism with AD, we used the key search terms including “*MEF2C*,” “rs190982,” “Alzheimer's disease,” and “AD,” combined with Boolean operators as appropriate to conduct a systematic literature search in PubMed, EMBASE, and the Cochrane library for publications up to December 2015. As to the limitation of language, only English publications were qualified. Additional studies were acquired from the reference lists of relevant primary articles. Finally, we adopted fixed-effects inverse variance-weighted methods to combine our results with the data from meta-analysis of 74,046 individuals [14] and other reports on the associations between *MEF2C* (rs190982) and LOAD [15–17]. At the same time, we generated I^2^ to estimate the possible influence of study heterogeneity on our results. Stata V.12.0 was used to perform all these analyses.
